# Editorial: Skeletal muscle in age-related diseases: From molecular pathogenesis to potential interventions

**DOI:** 10.3389/fphys.2022.1056479

**Published:** 2022-10-17

**Authors:** Michelle S. Parvatiyar, Rizwan Qaisar

**Affiliations:** ^1^ Department of Nutrition and Integrative Physiology, Florida State University, Tallahassee, FL, United States; ^2^ Department of Basic Medical Sciences, College of Medicine, University of Sharjah, Sharjah, United Arab Emirates

**Keywords:** sarcopenia, skeletal muscle, aging, oxidative stress, senescence

## Introduction

Skeletal muscle is a dynamic organ that responds to various stimuli by altering its size, strength, and composition. Maintenance of skeletal muscle mass and strength is critical for well-being and overall health. Conversely, ageing, and age-related diseases are associated with the loss of muscle mass and strength, termed sarcopenia. The exact molecular mechanisms of sarcopenia are poorly understood, which hamper the development of effective interventions to boost muscle mass and strength in ageing.

Sarcopenia is accompanied by functional disability and decreased quality of life. Skeletal muscle exhibits several characteristic alterations in sarcopenia, including diminished mitochondrial respiratory activity (Short et al., 2005) contractile dysfunction (Joseph et al., 2012; Grevendonk et al., 2021) elevated oxidative stress (Meng and Yu, 2010), reduced satellite cell count (Zwetsloot et al., 2013), senescence phenotype (Saito and Chikenji, 2021), loss of motor units (Piasecki et al., 2016), neuromuscular junction degradation (Karim et al., 2022), dysregulated calcium homeostasis (Qaisar et al., 2020), and hormonal changes (Qaisar et al., 2013).

Sarcopenia may be physiological and accompanied by healthy ageing without comorbidities. Nevertheless, sarcopenia is associated with alterations in skeletal muscle gene expression involving damage accumulation and compensatory mechanisms. An ambitious study by Tumasian III et al. investigated this question by extracting RNAs from muscle biopsies of healthy individuals over a wide age range. Altogether, 1,134 RNAs changed significantly with advancing age out of the 57,205 protein-coding and non-coding RNAs (Tumasian et al., 2021).

After its onset, sarcopenia is difficult to treat because it involves complex molecular interactions and is a risk factor for developing other diseases. Consequently, developing pharmaceutical strategies for sarcopenia remains a critical challenge due to multifactorial etiology and interface of skeletal muscle with multiple organs in the context of ageing. In our Research Topic “*Skeletal Muscle in Age-Related Diseases: From Molecular Pathogenesis to Potential Interventions*” several studies address important questions in the field as well as examine potential molecular targets to treat ageing skeletal muscle.

During skeletal muscle ageing, mitochondrial dysfunction contributes to reactive oxygen species (ROS)-induced oxidative damage (Qaisar et al., 2018), while exercise can improve mitochondrial function and reduce oxidative stress (Holloszy, 1967). However, this therapeutic effect of exercise is gradually blunted with advancing age, partly due to limiting exercise capacity and related health constraints. Therefore, understanding the molecular underpinnings of oxidative stress pathways and developing strategies to circumvent them may provide new bedside therapeutic options that are not reliant on exercise alone.

Ruan et al. examined the role of the long non-coding RNA (lncRNA) metastasis-associated lung adenocarcinoma transcript 1 (MALAT1), which was found to have decreased expression in aged skeletal muscle. Previous studies have suggested that changes in lncRNA expression can influence muscle growth and differentiation by incompletely understood mechanisms (Butchart et al., 2016). While it is known that lncRNAs can function as sponges that regulate levels of free microRNAs (miRNAs), the study by Ruan et al. examined their role in the less understood realm of muscle ageing. Previously the authors showed that heightened ROS production associated with ageing is accompanied by increased senescence-associated miRNA-34a-5p levels (Fulzele et al., 2019). In the current study, skeletal muscles obtained from young and aged mice of both sexes were examined for reciprocal changes in MALAT1 and miR-34a-5p expression. *In-vitro* studies in C_2_C_12_ myoblasts demonstrated that H_2_O_2_ decreased MALAT1 expression, while siRNA-mediated MALAT1 knock-down significantly increased TGF-β1 expression. The crosstalk or interplay between oxidative conditions/MALAT1/miR-34a may represent a missing link connecting ageing phenomenon in skeletal muscle with the development of fibrosis and cellular senescence. Studies such as this may unlock new potential age-related therapeutic targets to counter the deterioration of muscle health.

The study by You and Chen directly addressed the effect of age-associated motor neuron loss on the development of sarcopenia. The authors observed that aged mice with obvious signs of sarcopenia were better protected from accelerated denervation-induced muscle atrophy seen in much younger mice. The overall skeletal mass is dictated by factors that determine the net balance between the availability of its building blocks. e.g., protein synthesis (anabolic) versus degradation (catabolic). Interestingly, in aged mice, the tibialis anterior (TA) muscle displayed resistance to denervation-induced atrophy. It was found that aging promotes protein synthesis rates and ribosomal RNA (rRNA) biogenesis in denervated TA, which is independent of mTORC1 regulation but *via* enhanced signaling through the Akt-GSK-3β pathway.

Another line of thought in treating sarcopenia in aged individuals is replacing molecular factors lost during aging. While iTRAQ analysis yielded a lengthy list of peptide changes, the study by Lo et al. cut to the chase and first examined whether fetal skeletal muscle extract could benefit muscle health of aged mice. After 8 weeks of treatment, the aged mice showed enhanced muscle performance with increased lean mass, heightened grip strength, and isometric force generation. While the authors have not yet identified the factors in the fetal serum providing beneficial effects on aged muscle, the study is intriguing. Further analysis may yield one or multiple curative factors that can be replaced by direct injection or other administration methods to treat sarcopenia.

Exercise training accompanied by nutritional approaches remains the best non-pharmaceutical treatment strategy to counter sarcopenia. However, in the elderly or immobilized, these may not be plausible treatment strategies. Falqueto et al. highlight the pros and cons of using anabolic-androgenic steroids to increase muscle function and mass. Sarcopenia is driven by diverse pathological processes in ageing patients, including elevated muscle catabolism than anabolism. The combination of anabolic hormones and exercise training can reverse muscle wasting in aged individuals. However, in some studies, the type of exercise mattered—with resistance training yielding favorable results by minimizing fatigue and exercise intolerance while increasing muscle strength, hypertrophy, and power. Although these above outcomes appear favorable, the discernable observed may depend on differences in participant exclusion criteria. Overall, it is promising that administration of anabolic hormones to some patients bolstered the exercise effect on sarcopenia, at least in some patients.

Experimental studies highlighted in this edition provide new and provocative findings that represent novel targets and potential as alternative therapies for sarcopenia. These studies further our understanding of the role of lncRNAs in protecting against muscle senescence, the effect of innervation and ageing, and beneficial properties of fetal muscle extract in sarcopenic muscle.
